# Spatiotemporal Visualization of Insecticides and Fungicides within Fruits and Vegetables Using Gold Nanoparticle-Immersed Paper Imprinting Mass Spectrometry Imaging

**DOI:** 10.3390/nano11051327

**Published:** 2021-05-18

**Authors:** Run Qin, Ping Li, Mingyi Du, Lianlian Ma, Yudi Huang, Zhibin Yin, Yue Zhang, Dong Chen, Hanhong Xu, Xinzhou Wu

**Affiliations:** 1 State Key Laboratory for Conservation and Utilization of Subtropical Agro-Bioresources and Key Laboratory of Natural Pesticide and Chemical Biology of the Ministry of Education, South China Agricultural University, Guangzhou 510642, China; qinrun1995@stu.scau.edu.cn (R.Q.); liping08@stu.scau.edu.cn (P.L.); dmy626@stu.scau.edu.cn (M.D.); mll98@stu.scau.edu.cn (L.M.); huangyd@stu.scau.edu.cn (Y.H.); zy3333@stu.scau.edu.cn (Y.Z.); cd2023001@stu.scau.edu.cn (D.C.); 2 Agro-Biological Gene Research Center, Guangdong Academy of Agricultural Sciences, Guangzhou 510640, China

**Keywords:** gold nanoparticles, paper imprinting, mass spectrometry imaging, food safety, pesticides, spatiotemporal visualization

## Abstract

Food safety issues caused by pesticide residue have exerted far-reaching impacts on human daily life, yet the available detection methods normally focus on surface residue rather than pesticide penetration to the internal area of foods. Herein, we demonstrated gold nanoparticle (AuNP)-immersed paper imprinting mass spectrometry imaging (MSI) for monitoring pesticide migration behaviors in various fruits and vegetables (i.e., apple, cucumber, pepper, plum, carrot, and strawberry). By manually stamping food tissues onto AuNP-immersed paper, this method affords the spatiotemporal visualization of insecticides and fungicides within fruits and vegetables, avoiding tedious and time-consuming sample preparation. Using the established MSI platform, we can track the migration of insecticides and fungicides into the inner region of foods. The results revealed that both the octanol-water partition coefficient of pesticides and water content of garden stuffs could influence the discrepancy in the migration speed of pesticides into food kernels. Taken together, this nanopaper imprinting MSI is poised to be a powerful tool because of its simplicity, rapidity, and easy operation, offering the potential to facilitate further applications in food analysis. Moreover, new perspectives are given to provide guidelines for the rational design of novel pesticide candidates, reducing the risk of food safety issues caused by pesticide residue.

## 1. Introduction

Virtually all agricultural products around the world are exposed to agrochemicals, such as insecticides and fungicides, and the ever-increasing use of agrochemicals has resulted in detrimental effects on human health, especially because of food safety issues [1,2]. Although pesticides play a vital role in preventing pest damage and bacterial decay, as well as ensuring food production during planting and after harvest, it is apparent that food and safety are the first priority [3]. With this in mind, maximum residue limits (MRLs), ranging from tens or hundreds of µg/kg to several mg/kg, have been established for various pesticides used on foods [4]. To simplify the onsite screening process and shorten the analysis time, surface-sampling-based analytical strategies have been established, especially in security check points [5,6]. However, there remains a blind spot in the ability to detect the penetration of pesticides into the interior of fruits and vegetables, which might lead to serious food safety issues and even the consumption of contaminated foods by the public. Moreover, relatively little is known about the absorption, distribution, metabolism, and elimination (ADME) of agrochemicals in fruits and vegetables consumed daily, which is of the utmost importance for the scientific assessment of the effect of pesticides on food safety. Therefore, developing a rapid and visualized strategy for tracking the spatiotemporal trajectory of pesticides residing within garden stuffs is of great concern for managing food safety.

Traditionally, pesticide detection can be achieved by gas chromatography or liquid chromatography coupled to mass spectrometry (GC-/LC-MS) [7]. Despite the excellent detection limit, chromatographic methods refer to stochastic average contents masked by bulk measurements, resulting in a loss of information on the spatiotemporal distribution of pesticides in fruits and vegetables. Thus, unceasing efforts have been made to develop visually based methods for tracking pesticide residues in the temporal and spatial dimensions. Fluorescence spectrometry [8] and isotope-labeling imaging techniques [9] have proven to be robust tools for monitoring pesticide distribution, although the perturbed distribution of pesticides derived from fluorescent tags and time-consuming pretreatment cannot be ignored. Another particularly promising approach is using Raman spectroscopy to map pesticide penetration in plant leaves. Benefiting from in situ detection and little sample preparation, this technique allows for the mapping of pesticides on the surface of tomato plants and their penetration and persistence in basil leaves [10,11]. Mass spectrometry imaging (MSI) has emerged as a unique technique for visualizing the spatiotemporal evolution of pesticides within biological tissues, with the advantages of label-free and nonspecific detection [12,13]. Among the available techniques, desorption electrospray ionization (DESI) can be used to monitor pesticide distribution both on the surface of leaves and in the cross section of plant stems [14]. Alternatively, laser ablation electrospray ionization (LA-ESI) has been reported for macroscopic and microscopic imaging of mycotoxins on fruits [15,16]. Although direct molecular imaging for fruit and vegetable tissues can be achieved using these techniques, the limited spatial resolution and uneven surfaces might compromise their performance and result in imaging artifacts [17]. Because of the unique features of high spatial resolutions ranging from microscale to submicroscale, laser-based sampling techniques, such as matrix-assisted laser desorption/ionization (MALDI) MSI [18,19,20] and laser desorption/ionization (LDI) MSI [21,22,23], have been valuable analytical tools in tissue and single-cell imaging. Hoever, in some cases, imaging artifacts might also occur, originating from matrix interference peaks in the low *m/z* range, inhomogeneous matrix deposition, and uneven sample surfaces [24,25].

Sample preparation is of principal importance in MSI analysis, especially for plant tissues [26,27]. One common practice for animal tissues, roots and stems of plant tissues, and even fruit and vegetable tissues, is histological sectioning. Using this strategy, Taira et al. and Pereira et al. demonstrated the utility of MALDI-MSI for monitoring the temporal distribution of fungicide residues (i.e., procymidone and imazalil) [15,28]. Despite being widely used, histological sectioning is incompatible with tissues that cannot be sliced, such as leaves and petals, and samples that may shrink and break, especially water-rich fruits and vegetables. To overcome these limitations, imprinting-based sample preparation strategies have received increasing attention for plant imaging because of their fragile tissues and uneven surfaces [29]. After imprinting infected potato sprouts onto tape, Tata et al. used DESI-MSI to monitor the fluctuation in glycoalkaloids present in sprouted potatoes, indicating pathogen invasion [30]. Enomoto et al. developed a through-hole alumina membrane (DIUTHAME) chip as a blotting material for mapping metabolites in strawberry fruit [31]. Moreover, gold nanoparticle multilayer and graphene oxide-based substrates have been proposed for small-molecule analysis [32,33,34]. Despite the successful application of these imprinting materials in food imaging, they still suffer from several problems when integrated with laser-based MSI analysis, such as a low laser absorption efficiency and surface charge accumulation. Recently, we developed gold nanoparticle (AuNP)-immersed paper as an imprinting material coupled to a built in-house LDI-TOFMS platform, revealing the uptake and translocation mechanisms of carrier-mediated pesticides in plants [35]. However, the general applicability and the potential of this AuNP-immersed paper imprinting strategy for fruit and vegetable imaging has not yet been explored.

Chlorantraniliprole and azoxystrobin, representing typical insecticides and fungicides, have been acknowledged for preventing pests and rot in the growing and postharvest periods, respectively [36]. Herein, we report the development of an AuNP-immersed paper imprinting MSI platform for acquiring the spatiotemporal migration of agrochemicals in various fruits and vegetables, such as apple, cucumber, pepper, plum, carrot, and strawberry. With the integrated advantages of a strong liquid absorption capacity of filter paper for fruit and vegetable tissues and the improved ionization efficiency induced by AuNPs, it affords a more intuitive visualization of whether agrochemical molecules enter fruits and vegetables, as well as the corresponding migration behaviors. Interestingly, the MSI results revealed that the migration rate of agrochemicals can differ with that of the agrochemical species, suggesting that the nature of pesticides themselves will affect their penetration into fruits and vegetables. More specifically, the octanol-water partition coefficient of pesticides and the water content of fruits and vegetables can influence the migration behaviors of pesticides into foods. Taken together, these results demonstrate the successful application of the AuNP-immersed paper imprinting technique as a viable option for tracking pesticide migration in fresh fruits and vegetables, facilitating more precise food safety evaluations.

## 2. Materials and Methods

### 2.1. Chemicals

Chloroauric acid (HAuCl_4_, >98% purity) and sodium citrate (>98% purity) were purchased from Energy Chemistry Ltd. (Shanghai, China). Chlorantraniliprole and azoxystrobin were purchased from J&K Scientific Ltd. (Beijing, China). In addition, qualitative and quantitative filter papers with different filtration rates were all purchased from Hangzhou Wohua Filter Paper Ltd. (Hangzhou, China). The apples, cucumbers, peppers, plums, carrots, and strawberries were bought from a local supermarket.

### 2.2. Characterization and Measurements

The morphology of the AuNPs and AuNP-immersed paper were characterized using a scanning electron microscope (SEM; FEI Verios 460, Thermo Fisher, Hillsboro, CA, USA) and a transmission electron microscope (TEM; FEI Talos F200S, Thermo Fisher, Hillsboro, CA, USA). The UV–VIS absorption spectrum of the synthesized AuNPs were obtained using a UV–VIS spectrophotometer (UV-5500PC, Shanghai Yuanxi Instrument Ltd., Shanghai, China). The results of the pesticides within the apple tissues were acquired by high performance liquid chromatography (HPLC; Agilent 1290B, Santa Clara, CA, USA).

### 2.3. Preparation of AuNPs, AuNP-Immersed Paper, and Pesticides

AuNPs were synthesized according to previous reports, with some modifications [37]. Briefly, the stock solution comprises 0.5 g HAuCl_4_ dissolved in 25 mL deionized water. In addition, a 0.01% HAuCl_4_ solution was prepared by diluting 1 mL stock solution to 100 mL. Two milliliters of 1% sodium citrate was added to the solution with high-speed stirring once it bubbled. Then, it was cooled to room temperature after 10 min of refluxing at the boiling point and was stirred continuously for 15 min. Finally, the AuNP suspension was stored at 4 °C until use. For the preparation of the AuNP-immersed paper, we immersed the filter paper in a petri dish containing an AuNP solution for 20 min and then air dried it. Individual stock solutions of chlorantraniliprole and azoxystrobin at a concentration of 1000 mg/L were first dissolved in acetonitrile. Afterwards, the stock solutions were sequentially diluted with deionized water to a concentration of 1 × 10^−5^ mol/L before the MS experiment.

### 2.4. Quantitative Determination of Chlorantraniliprole by HPLC

A 10 g apple sample was dissolved in 15 mL acetonitrile in a 50 mL centrifuge tube, placed in a vortex mixer for 5 min, and extracted ultrasonically at 30 °C for 30 min. Then, 5 g anhydrous magnesium sulfate and 2 g sodium chloride were added to a 50 mL centrifuge, shaken vigorously for 1 min, and centrifuged at 5000 r/min for 10 min. The supernatant was concentrated to the required purification using a 0.22 µm PTFE membrane, and then 3 mL supernatant was dried by rotary evaporation. It was diluted to 150 µL acetonitrile ultrasonically at 30 °C for 30 min and prepared for HPLC analysis. The ratio of the mobile phase for the chlorantraniliprole analysis was equal proportions of acetonitrile and water (*v*/*v*, 50/50), and that for the azoxystrobin mobile phase was 65% acetonitrile and 35% water. The analytical column employed was a Zorbax Stablebond C18 column (250 mm × 4.6 mm, 5 μm; Agilent). The flow rate of the column was 1.0 mL/min for chlorantraniliprole, and the column temperature was set at 25 °C. The detection wavelength of the UV detector was 210 nm for chlorantraniliprole.

### 2.5. Laser Desorption/Ionization (LDI) Source

A built in-house reflection time-of-flight mass spectrometer was built as reported earlier, with a few modifications (see Figure S1 in the Supplementary Materials) [12,35]. Briefly, a 355 nm laser with a 5 ns pulse duration and a 10 Hz repetition rate (Minilite II, Continuum Inc., West Newton, MA, USA) was adopted in this study. Given the large sampling area of fruit and vegetable tissues, the laser spot was ill-focused to ~120 µm with an aspheric lens (f = 79.0 mm, NA = 0.143, AR coated: 350–700 nm, Thorlabs Inc., Newton, NJ, USA). This resulted in a trade-off between a desirable imaging resolution and a reasonable analysis time. Unless otherwise stated, the laser energy could be set as low as 20 μJ for the MSI experiments because of the improved ionization efficiency of the AuNP-immersed paper. Additionally, although higher laser irradiance affords more detectable molecular species, the mass spectral resolution can be compromised because of the high energy dispersal of ions. Therefore, a time-lag focusing technique derived from a multichannel delay pulse generator was adopted for improved spectral resolution [38,39]. The region of interest (ROI) can be clearly observed by a built-in camera. The MSI results were acquired by pixel-by-pixel laser irradiation of the imprinted food pattern.

### 2.6. Time-of-Flight Mass Spectrometer

Ions generated from manual stamping of fruits and vegetables on AuNP-immersed paper were pulled into the acceleration region with a pulsed extraction voltage. The ions entered a double-stage reflector and arrived at the microchannel plate (MCP) detector. The AuNP-assisted LDI source and TOF mass analyzer are located in the same vacuum chamber under a pressure of 1 × 10^−5^ Pa. A high-precision two-dimensional positioner stage (OSMS20-85, Sigma Koki, Tokyo, Japan) with a maximum travel distance and movement speed of 85 mm and 25 mm/s was adopted for the sample scanning and imaging.

### 2.7. Date Processing for MSI

A digital oscilloscope (WaveRunner 6100A, LeCroy, New York, NY, USA) with a 5 G/s sampling rate and 1 GHz analog bandwidth was used for recoding the ion signals. The output signals from the oscilloscope were processed using a self-compiled program written in LabVIEW (National Instruments, Austin, TX, USA). Ultimately, the mass spectra were obtained by Origin 9 software (OriginLab, Northampton, MA, USA), and MS images derived from ions of interest were obtained by Surfer 9 software (Golden Software, Inc., Golden, CO, USA) [21,35,40].

### 2.8. Workflow for the AuNP-Immersed Paper Imprinting MSI Platform

**Figure 1:**
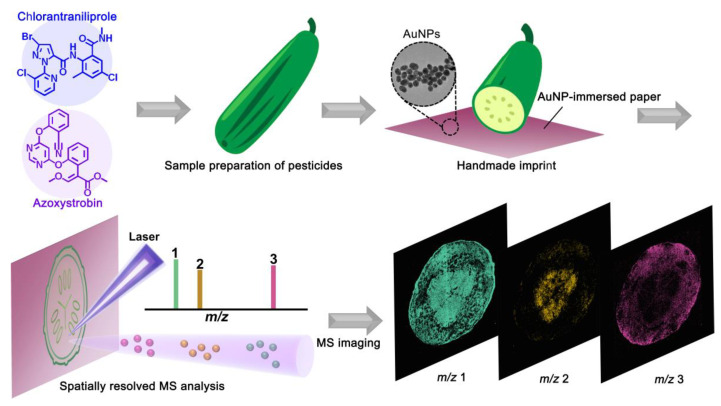
AuNP-immersed paper imprinting mass spectrometry imaging (MSI) platform and workflow. All fruits and vegetables were imprinted on AuNP-immersed paper using manual stamping for the MSI analysis.

As shown in Figure 1, the individual pesticide solutions were sprayed onto fruits and vegetables, which were then naturally air-dried. All of the control groups were kept away from the pesticide treatment. For rapid analysis, all of the fruits and vegetables (apples, cucumbers, peppers, plums, carrots, and strawberries) were cut apart using a knife, and one side was imprinted on AuNP-immersed paper with manual stamping for the MSI analysis. Then, conductive graphite was used to adhere the AuNP-immersed paper to the sampling plate. MSI results were acquired by pixel-by-pixel laser irradiation of the imprinted food pattern. Additionally, each pixel was normalized to the base pixel for the MS images with the highest signal intensity per image. A pixel size of 120 µm and three pulses for each pixel were adopted for each image, unless otherwise stated.

## 3. Results and Discussion

### 3.1. Improved Desorption/Ionization Efficiency of Pesticides Using AuNP-Immersed Paper

To demonstrate the feasibility of AuNP-immersed paper-based MSI for the rapid detection of agrochemicals within foods, the improved desorption/ionization efficiency was first investigated by spotting pesticides on stainless steel plates with AuNPs and without AuNPs. As shown in Figure S2 (Supplementary Materials), even though Na^+^/K^+^ contaminants were hardly observed in the LDI spectrum (blank line in Figure S2 in the Supplementary Materials), strong signals of chlorantraniliprole and azoxystrobin with a high signal-to-noise ratio were observed using AuNP-assisted LDI (red line in Figure S2 in the Supplementary Materials). One issue to note is that both pesticides were detected in the form of [M+Na]^+^ instead of [M+H]^+^, which can be ascribed to the fact that 1% sodium citrate was essential in the AuNP synthesis as a capping and reducing agent. To further elucidate the feasibility of AuNP-immersed paper for food imaging, the typical mass spectra of both pesticides spotted on AuNP-immersed paper were also acquired (see Figure S3 in the Supplementary Materials). With the nanosized feature of AuNPs embedded in paper tissues, the mass spectrum of chlorantraniliprole was dominated by [M+Na]^+^, [M+K]^+^, and two characteristic fragments (i.e., [M-C_9_H_9_N_2_OCl]^+^ and [M-CH_3_NH]^+^), as observed in Figure S3a (Supplementary Materials). The strong preference for the formation of fragmented ions rather than alkali metal adducts possibly originated from the fragile structure of chlorantraniliprole under higher laser irradiance. Given that the signal intensity of [M-C_9_H_9_N_2_OCl]^+^ was much stronger than that of the other peaks, it was selected for the subsequent MSI analysis. In contrast, strong [M+Na]^+^ signals can be obtained for azoxystrobin (see Figure S3b in the Supplementary Materials). In addition, the fragment ion at *m/z* 372.1 can be assigned to [M-CH_3_O]^+^ to azoxystrobin. The fragmentation pathways of chlorantraniliprole and azoxystrobin are shown in Figures S4 and S5 (Supplementary Materials), respectively. Taken together, this matrix-free strategy affords great potential in the sensitive detection of various pesticides with improved ionization yields.

### 3.2. Characterization of AuNPs on Immersed Filter Paper

**Figure 2:**
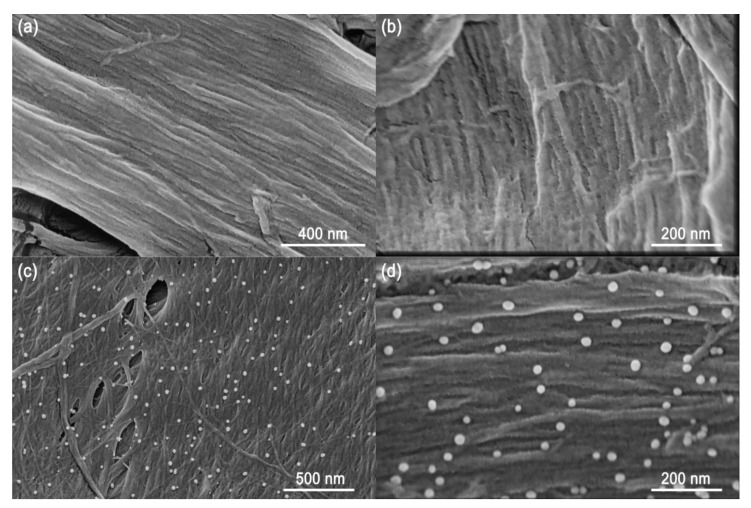
SEM images of original filter paper without AuNP solution immersion at (**a**) 100,000× and (**b**) 200,000× magnification, and AuNP-immersed filter paper at (**c**) 80,000× and (**d**) 200,000× magnification, respectively.

Matrix homogeneity is of crucial importance for MSI analysis, avoiding matrix-related signal fluctuation and imaging artifacts. Poor reproducibilities for pixel-to-pixel, region-to-region, and sample-to-sample analyses commonly arise as a result of inhomogeneous sample/matrix crystals. Before AuNP-immersed paper imprinting MSI analysis for fruit and vegetable tissues, we first investigated the homogeneity of the AuNPs imbedded onto filter paper. Compared to the original filter paper (Figure 2a,b), AuNPs can be imbedded into the microstructural fiber uniformly after solution immersion (Figure 2c,d). The integrated results from the SEM and TEM images (see Figure S6 in Supplementary Materials) revealed that the mean particle diameter of AuNPs was ~27 nm, which affords a strong UV–VIS absorption in the length of 355 nm, resulting in improved laser desorption and energy transfer (see Figure S7 in Supplementary Materials). Collectively, given that the typical laser spot in the MSI analysis normally ranges from several to dozens of micrometers, this paper imprinting strategy with a nanoscale AuNP deposition can offer pixel-to-pixel signal enhancement without imaging artifacts.

### 3.3. Spatiotemporal Distribution of Insecticides Using AuNP-Immersed Paper Imprinting MSI

**Figure 3:**
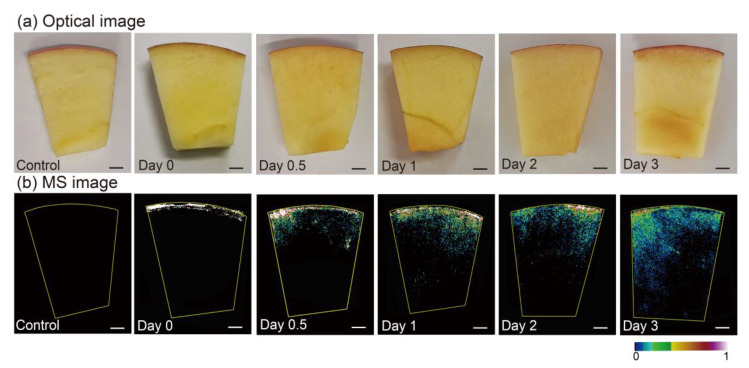
(**a**) Optical images and (**b**) the corresponding MS images of chlorantraniliprole in apple slices without chlorantraniliprole application and with 0, 0.5, 1, 2, and 3 days after chlorantraniliprole spraying. Scale bar of 4 mm.

Chlorantraniliprole has been widely used in fruits and vegetables as an excellent insecticide, owing to its remarkable selectivity and low toxicity to mammals [41]. Previous reports have indicated that insecticides are persistent on food surfaces [42], yet few studies have focused on whether and how fast they enter the interior of foods. To demonstrate the superior ability of this analytical method for the spatiotemporal visualization of pesticide migration in fruits, several apples were sprayed with chlorantraniliprole and stored at room temperature for several days before MSI analysis (Figure 3a). Then, they were cut apart and manually stamped onto AuNP-immersed paper. As shown in Figure 3b, the spatial distribution of chlorantraniliprole within apple slices at different times was acquired using MSI. In sharp contrast, no chlorantraniliprole signals were detected for the control apple, suggesting that the experimental procedure did not cause contamination of the samples. Interestingly, in the apple sprayed 20 min after pesticide application (corresponding to day 0), that chlorantraniliprole was distributed only in the pericarp region, and did not reach the flesh region. As time went by, the MS images of the apple slices 0.5 days after pesticide application revealed that chlorantraniliprole started migrating to the flesh region through the pericarp. More chlorantraniliprole molecules migrated deeper into the flesh region 1 day after pesticide application. Of particular interest is the finding that most pesticides still remained in the pericarp region even after 24 h. When chlorantraniliprole remained on the apple surface for 2 days, pesticides could migrate further, even reaching the apple core, and almost all the apple slices were distributed on day 3. It should be noted that the pesticide did not penetrate into the kernels of the apples with a uniform velocity, and the penetration speed of chlorantraniliprole on day 3 was faster than that on day 2. Considering the reduced signal intensity of chlorantraniliprole in the pericarp region on day 3, this phenomenon can be foreseen because more pesticides migrated into the pulp.

Moreover, the quantitative LC results indicated that the changes in the pesticide contents in the peel and pulp were consistent with the MSI results (see Figure S8 in the Supplementary Materials). One issue to note is that most pesticides first resided in the peel after pesticide application, and did not penetrate into the fruit pulp immediately. Integrating the MSI and LC results indicates that, although the whole amount of chlorantraniliprole remained unchanged, the concentration of chlorantraniliprole within the apple kernel gradually increased over time, whereas that on the apple pericarp decreased. Given that pesticides might penetrate fruits after a certain amount of time and that most food safety inspections are dependent on homogenization and surface analysis, inaccurate results may be obtained.

### 3.4. Spatiotemporal Distribution of Fungicides within Strawberry and Cucumber Tissues Using AuNP-Immersed Paper Imprinting MSI

**Figure 4:**
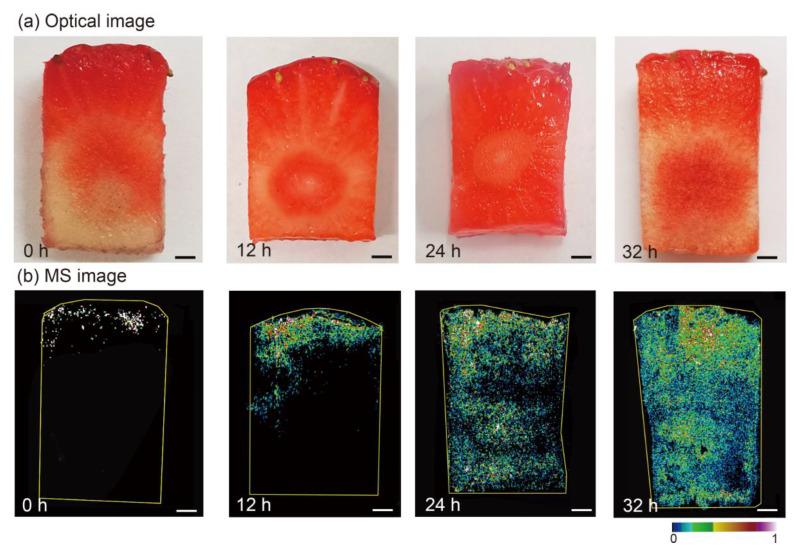
(**a**) Optical images and (**b**) the corresponding MS images of azoxystrobin in strawberry slices 0 h, 12 h, 24 h, and 32 h after being sprayed with azoxystrobin. Scale bar, 3 mm.

Fungicides, representing common preservatives, have been widely used in fruits and vegetables to improve preharvest yield and quality and to reduce postharvest decay. Among them, azoxystrobin has been widely used in fruits and vegetables, such as strawberries and cucumbers, to enhance yield and quality [43]. The dynamic spatiotemporal evolution of azoxystrobin within strawberry tissues, ranging from 0 h (sprayed 20 min after pesticide application) to 32 h after application, can be clearly seen in Figure 4. The spatiotemporal distribution of pesticides within the strawberry tissues could be acquired even when the strawberry was soft and easily crushed during manual stamping. It should be noted that azoxystrobin did not penetrate the strawberries at a uniform velocity, as observed 12 h after application. Of particular interest was the finding that azoxystrobin was evenly distributed in the strawberry slices 24 h after application. It can be inferred that there are some disadvantages for traditional pesticide residue detection, that the selection of different locations may result in incorrect information. More specifically, pesticides might have entered the fruits and vegetables even though the residual concentration of pesticides on the surface did not surpass the MRLs detected by conventional methods. Taken together, the MSI results revealed that pesticides spreading into the food interior can still cause great concern for food safety, and pesticides can more easily enter the interior of fruits and vegetables without peels.

**Figure 5:**
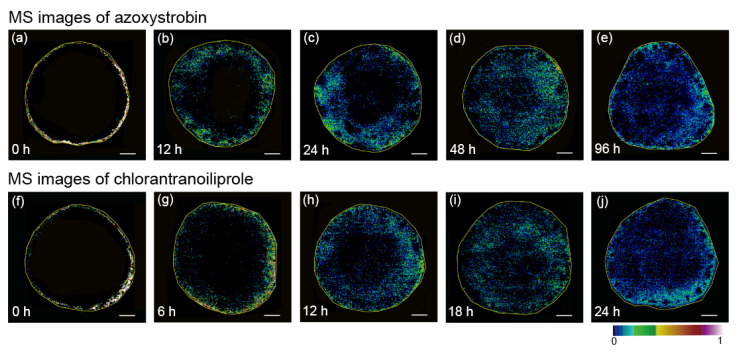
MS images of azoxystrobin (**a**) 0 h, (**b**) 12 h, (**c**) 24 h, (**d**) 48 h, and (**e**) 96 h after application. MS images of chlorantraniliprole (**f**) 0 h, (**g**) 6 h, (**h**) 12 h, (**i**) 18 h, and (**j**) 24 h after application. Scale bar of 5 mm.

Additionally, the dynamic spatial evolution of azoxystrobin within cucumber tissues was acquired. A cucumber slice was divided into three parts (i.e., the pericarp, flesh, and seed cavity), and the corresponding optical image and imprinted images before and after MSI measurement of cucumber are shown in Figure S9 (Supplementary Materials). It should be noted that azoxystrobin migrated to the flesh region 12 h after azoxystrobin application. Then, azoxystrobin started migrating to the seed cavity, and the pesticide was not distributed throughout the center region until 48 h after application, as shown in Figure 5b–e. It should also be noted that the pesticide distribution ranged from a small amount of the seed cavity to almost the entire seed cavity; thus, azoxystrobin migrated more slowly in the seed cavity than in the flesh region. This phenomenon can be ascribed to the fact that the seed cavity contains seeds, making it difficult for pesticides to migrate. Therefore, surface-sampling-based procedures applied at security check points are not sufficient, even though surface sampling analysis can simplify the onsite screening process and shorten the analysis time. Many pesticides remaining on the peels of fruit can be detected by traditional surface-sampling-based analytical strategies, yet the pesticides could distribute uniformly within the fruit and vegetable tissues after a period of time, especially for fruits and vegetables with thin or no peels, such as strawberries. As shown in Figure 4 and Figure 5a–e, the migration speed of azoxystrobin within the strawberry tissues was faster than that within cucumber tissues. This is understandable, as strawberries do not have peels like cucumbers, resulting in pesticides having easier access to the interior of the fruits. In addition, the MS images showed azoxystrobin distribution in the pulp, as shown in Figure S10 (Supplementary Materials). Insights into the absorption, distribution, and kinetics of pesticides can be hindered by stochastic average values from bulk measurements; thus, the spatiotemporal distribution of pesticides within food tissues has fundamental significance in food safety assessments. In summary, AuNP-immersed paper imprinting MSI affords rapid, simple, and spatiotemporal visualization detection of fungicides within fruit and vegetable tissues, which is complementary to conventional detection methods.

### 3.5. Comparisons of Chlorantraniliprole and Azoxystrobin Migration Speed Using AuNP-Immersed Paper Imprinting MSI

Previous studies have proposed various methods for reducing pesticides on fruits and vegetables, such as washing, cooking, peeling, and ultrasound [44,45]. Recently, Ruengprapavut et al. investigated the effectiveness of chemical solutions (e.g., sodium bicarbonate, vinegar, and deionized water) on the removal of carbaryl residues from cucumber and chili presoaked in carbaryl [46]. Pesticides can penetrate through the pericarp and enter the interior of foods, yet the factors that affect the migration speed of pesticides within fruit and vegetable tissues are poorly understood. Encouraged by the above results, we further performed AuNP-immersed paper imprinting MSI to investigate the differing migration behaviors of chlorantraniliprole and azoxystrobin within cucumber tissues. The spatiotemporal evolution of chlorantraniliprole within cucumber tissues at different times after pesticide application (from 0 h to 24 h) can be clearly observed in Figure 5f–j. At 0 h, the MS image showed that chlorantraniliprole was distributed only on the pericarp region. Afterwards, it migrated quickly from the pericarp to the flesh region, whereas only a few pesticide molecules could be observed in the center region 12 h after application, as shown in Figure 5f–j. It should be noted that the pesticide was distributed in all of the cucumber slices 24 h after pesticide application. In sharp contrast, azoxystrobin migrated slower than chlorantraniliprole, as observed from the MS images (Figure 5a–e). Specifically, azoxystrobin did not migrate toward the inner region of cucumber tissues until 6 h after application, whereas it was distributed in all of the cucumber slices after 18 h.

### 3.6. The Potential Mechanism for Discrepant Migration Rate of Pesticides within Fruit and Vegetable Tissues

**Figure 6:**
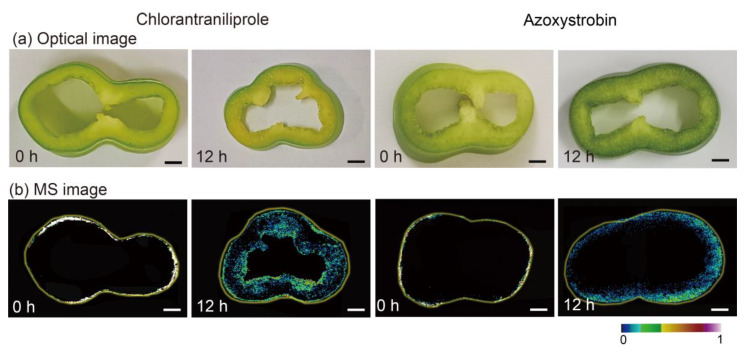
(**a**) Optical images and (**b**) MS images of pepper slices 0 h and 12 h after the application of chlorantraniliprole and azoxystrobin. Scale bar of 3 mm.

The above phenomenon is understandable, because these two pesticides afford different log *Kow* values, corresponding to the logarithm of the octanol-water distribution coefficient, which plays a vital role in the pesticide migration rate [47]. Interestingly, the residue levels in the processed and unprocessed products depended mostly on the physical and chemical characteristics of the residues, especially the octanol-water partition coefficient. Compounds with a log *Kow* value were more polar. The log *Kow* values of chlorantraniliprole and azoxystrobin were calculated with ACD/LogD suite v.14.0 software to be 3.6 and 5.7 at 25 °C, respectively, suggesting that chlorantraniliprole can be more polar than azoxystrobin [48]. It has been reported that the larger the log *Kow* value is, the stronger the lipophilicity and the easier the compound can be adsorbed on leaves containing a waxy layer [49]. Similarly, high polarity might contribute to the faster migration of the pesticide to the interior region, where the water content is high [15]. As observed from Figure 5, chlorantraniliprole had a higher migration rate than azoxystrobin within cucumber tissues becsuse of a higher log *Kow* value. These observations also proved the correlation between log *Kow* values of pesticides and the corresponding migration rate within food tissues. Furthermore, we also compared the migration behaviors of chlorantraniliprole and azoxystrobin within pepper tissues using AuNP-immersed paper imprinting MSI. As shown in Figure 6, it took only 24 h for chlorantraniliprole to migrate from the outer skin throughout the pepper, whereas azoxystrobin remained in the outer skin after the same amount of time post-application. The MSI results further support the fact that the log *Kow* values of pesticides play a vital role in the speed of migration. In addition, compared with the migration rate of chlorantraniliprole within apple tissues (Figure 3), azoxystrobin had a much lower migration rate (see Figure S11 in the Supplementary Materials). Of particular interest, considering that the water content of carrot is lower than that of cucumber, it took a much longer time (5 days) for chlorantraniliprole to uniformly distribute within the carrot tissues than the cucumber tissues (see Figure S12 in the Supplementary Materials). Understanding the migration rate and potential pesticide persistence within the interior region of food tissues is critical for assessing the associated food security, especially for pesticides with relatively high log *Kow* values and fruits and vegetables with relatively high water contents. Taken together, these findings provide novel insights into the migration rates of pesticides within food tissues, as determined by their log *Kow* values and intrinsic water content, and lay a solid foundation for the development of MSI-based visualization methods that are complementary to conventional pesticide residue detection.

## 4. Conclusions

In summary, we demonstrated a method for the spatiotemporal visualization of insecticides and fungicides within a variety of fruit and vegetable tissues using AuNP-immersed paper imprinting MSI. This material, with the synergistic characteristics of a strong tissue absorption capacity and improved ionization efficiency, can overcome the deficiencies of conventional imprinting materials, meeting the increasing analysis requirements for rapid, sensitive, and visualized detection of pesticide migration within fruits and vegetables in the temporal and spatial dimensions. Additionally, this approach exerts considerable effects on simplified sample preparation in MSI applications, providing a better scientific assessment of food safety and the effect of pesticides on fruits and vegetables consumed daily. The results presented here reveal that pesticides with larger log *Kow* values and fruits and vegetables with higher water contents might contribute to the faster migration speed of pesticides into foods. This might provide guidelines for the development of new pesticides with reduced detrimental effects caused by pesticide residues. Efforts will be made in the future to provide deeper insight into the various scientific issues regarding food safety, such as the ADME of agrochemicals in food tissues. Beyond that, numerous possibilities remain for multifunctional nanocomposites with synergistic effects [50,51] for tissue imprinting imaging, especially in life science and environmental science.

## Data Availability

All data are available in the manuscript and in the Supplementary Material.

## Supplementary Materials

The following are available online at https://www.mdpi.com/article/10.3390/nano11051327/s1. Figure S1: Schematic diagram of a laser desorption/ionization time-of-flight mass spectrometer. Figure S2: Comparative mass spectra of chlorantraniliprole and azoxystrobin using LDI-MS and AuNP-assisted LDI-MS. Figure S3: Mass spectra of chlorantraniliprole and azoxystrobin deposited on the AuNP-based filter paper. Figure S4: The typical fragmentation pathways of chlorantraniliprole. Figure S5: The typical fragmentation pathways of azoxystrobin. Figure S6: TEM image of the synthesized AuNPs. Figure S7: UV–VIS spectrum of the synthesized AuNPs. Figure S8: HPLC results of chlorantraniliprole concentration in the pericarp, flesh, and entirety of apples at different times after the application of chlorantraniliprole. Figure S9: Optical images of a cucumber slice sprayed with pesticide, and a AuNP-immersed filter paper imprint of a cucumber slice before and after laser irradiance. Figure S10: Optical image and corresponding MS images of a pulp 1 day and 2 days after azoxystrobin spraying. Figure S11: Optical and MS images of an apple slice 0 days and 1 day after azoxystrobin spraying. Figure S12: Optical and MS images of a carrot slice 2 days and 5 day after chlorantraniliprole spraying.
