# Supplementary material for: Spatiotemporal Visualization of Insecticides and Fungicides within Fruits and Vegetables Using Gold Nanoparticle-Immersed Paper Imprinting Mass Spectrometry Imaging
Source: Nanomaterials (Basel). 2021 May 18;11(5):1327. doi: 10.3390/nano11051327 (PMC8157356; doi:10.3390/nano11051327)
Supplement: Supplementary file 1 [file nanomaterials-11-01327-s001.zip › nanomaterials-1216015-supplementary.pdf]

nanomaterials

Article

Spatiotemporal Visualization of Insecticides and Fungicides within Fruits and Vegetables using Gold Nanoparticle-Immersed Paper Imprinting Mass Spectrometry Imaging

Run Qin [1,†], Ping Li [1,†], Mingyi Du [1], Lianlian Ma [1], Yudi Huang [1], Zhibin Yin [2,*], Yue Zhang [1], Dong Chen [1], Hanhong Xu [1,*] and Xinzhou Wu [1,*]

[1] State Key Laboratory for Conservation and Utilization of Subtropical Agro-Bioresources and Key Laboratory of Natural Pesticide and Chemical Biology of the Ministry of Education, South China Agricultural University, Guangzhou 510642, China; qinrun1995@stu.scau.edu.cn (R.Q.); liping08@stu.scau.edu.cn (P.L.); dmy626@stu.scau.edu.cn (M.D.); mll98@stu.scau.edu.cn (L.M.); huangyd@stu.scau.edu.cn (Y.H.); zy3333@stu.scau.edu.cn (Y.Z.); cd2023001@stu.scau.edu.cn (D.C.)

[2] Agro-Biological Gene Research Center, Guangdong Academy of Agricultural Sciences, Guangzhou 510640, China

* Correspondence: zbyin@agrogene.ac.cn (Z.Y.); hhxu@scau.edu.cn (H.X.); wuxz@scau.edu.cn (X.W.)

† The authors contributed equally to this work.

Citation: Qin, R.; Li, P.; Du, M.; Ma, L.; Huang, Y.; Yin, Z.; Zhang, Y.; Chen, D.; Xu, H.; Wu, X. Spatiotemporal Visualization of Insecticides and Fungicides within Fruits and Vegetables by Gold Nanoparticle-Immersed Paper Imprinting Mass Spectrometry Imaging. *Nanomaterials* **2021**, *11*, 1327. https://doi.org/10.3390/nano11051327

Academic Editor: Oscar Ramos; Manuela Pintado; Alessandra Braga Ribeiro; Carla Pereira; Jose M. Lagaron

Received: 25 April 2021

Accepted: 15 May 2021

Published: date

Publisher's Note: MDPI stays neutral with regard to jurisdictional claims in published maps and institutional affiliations.

Copyright: © 2021 by the authors. Submitted for possible open access publication under the terms and conditions of the Creative Commons Attribution (CC BY) license (https://creativecommons.org/licenses/by/4.0/).

Table of content

Figure S1. Schematic diagram of a laser desorption/ionization time-of-flight mass spectrometer.

Figure S2. Comparative mass spectra of chlorantraniliprole and azoxystrobin using LDI-MS and AuNP-assisted LDI-MS.

Figure S3. Mass spectra of chlorantraniliprole and azoxystrobin deposited on the AuNP-based filter paper.

Figure S4. The typical fragmentation pathways of chlorantraniliprole.

Figure S5. The typical fragmentation pathways of azoxystrobin.

Figure S6. TEM image of the synthesized AuNPs.

Figure S7. UV-Vis spectrum of the synthesized AuNPs.

Figure S8. HPLC results of chlorantraniliprole concentration in the pericarp, flesh, and entirety of apples at different times after the application of chlorantraniliprole.

Figure S9. Optical images of a cucumber slice sprayed with pesticide, and a AuNP-immersed filter paper imprint of a cucumber slice before and after laser irradiance.

Figure S10. Optical image and corresponding MS images of a pulp 1 day and 2 days after azoxystrobin spraying.

Figure S11. Optical and MS images of an apple slice 0 days and 1 day after azoxystrobin spraying.

Figure S12. Optical and MS images of a carrot slice 2 days and 5 day after chlorantraniliprole spraying.

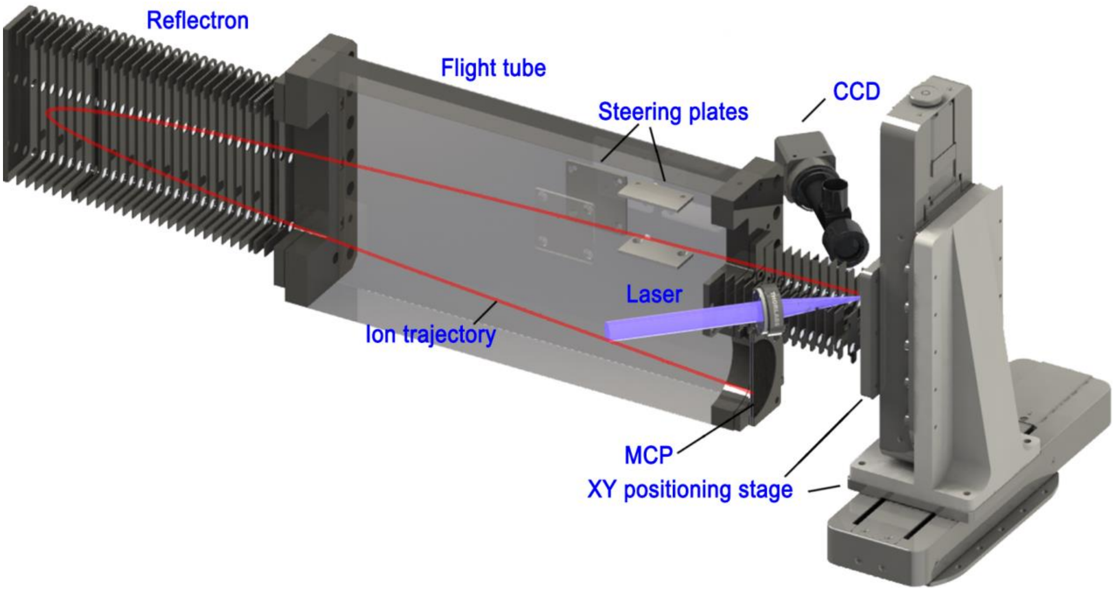

Figure S1. Schematic diagram of a laser desorption/ionization time-of-flight mass spectrometer.

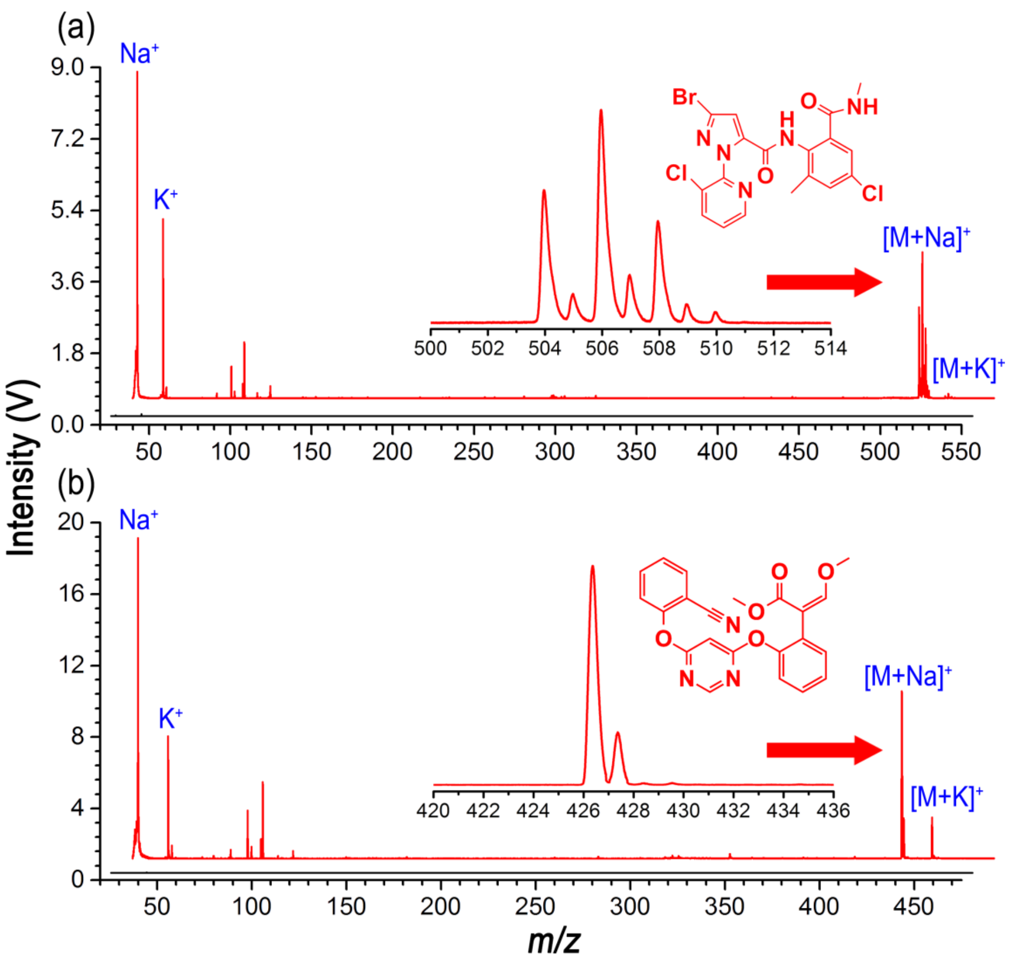

Figure S2. Comparative mass spectra of (**a**) chlorantraniliprole and (**b**) azoxystrobin using LDI-MS (black line) and AuNP-assisted LDI-MS (purple line). All the spectra were accumulated with 600 laser pulses. The insets indicate the isotope patterns and structures of the corresponding pesticide molecules.

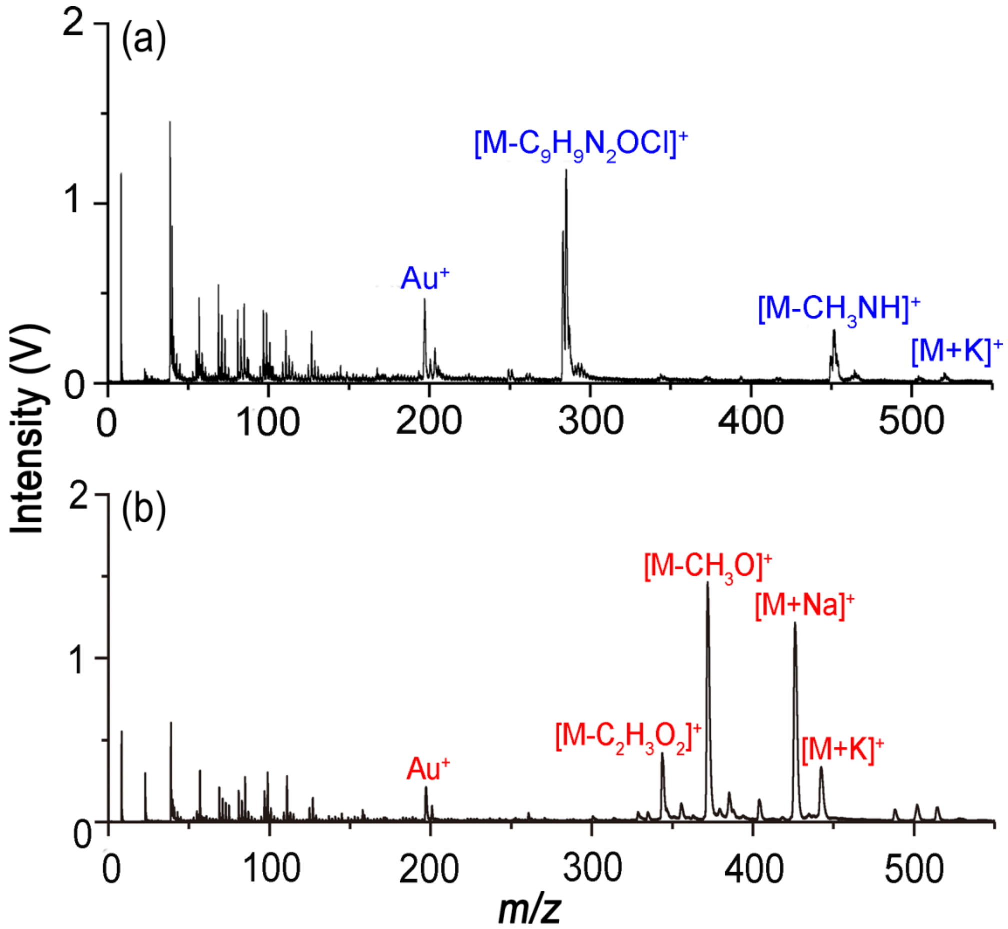

Figure S3. Mass spectra of (**a**) chlorantraniliprole and (**b**) azoxystrobin deposited on the AuNP-based filter paper. All the spectra were accumulated with 200 laser pulses.

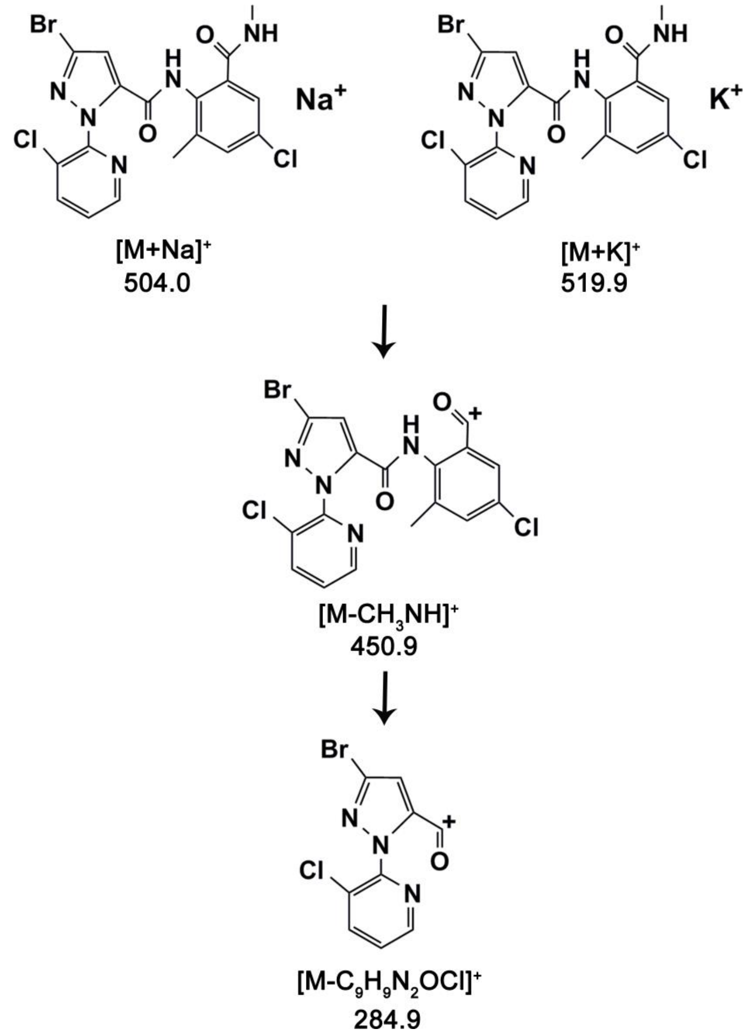

Figure S4. The typical fragmentation pathways of chlorantraniliprole for the production of the characteristic fragments, such as $[M\text{-}CH_3NH]^+$ at *m*/*z* 450.9 and $[M\text{-}C_9H_9N_2OCl]^+$ at *m*/*z* 284.9.

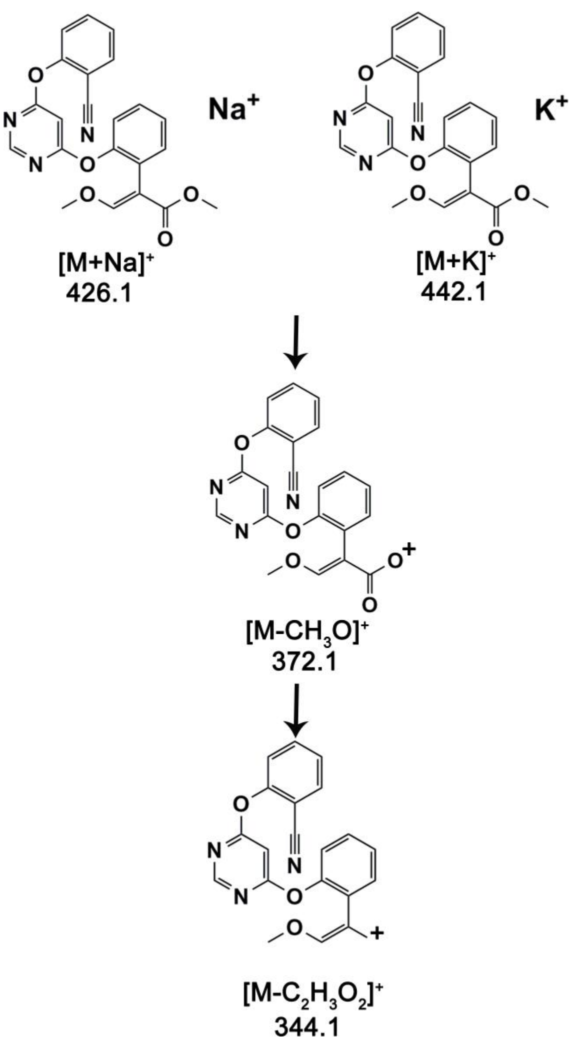

Figure S5. The typical fragmentation pathways of azoxystrobin for the production of the characteristic fragments, such as [M-CH_3O]$^+$ at *m/z* 372.1 and [M-$C_2H_3O_2$]$^+$ at *m/z* 344.1.

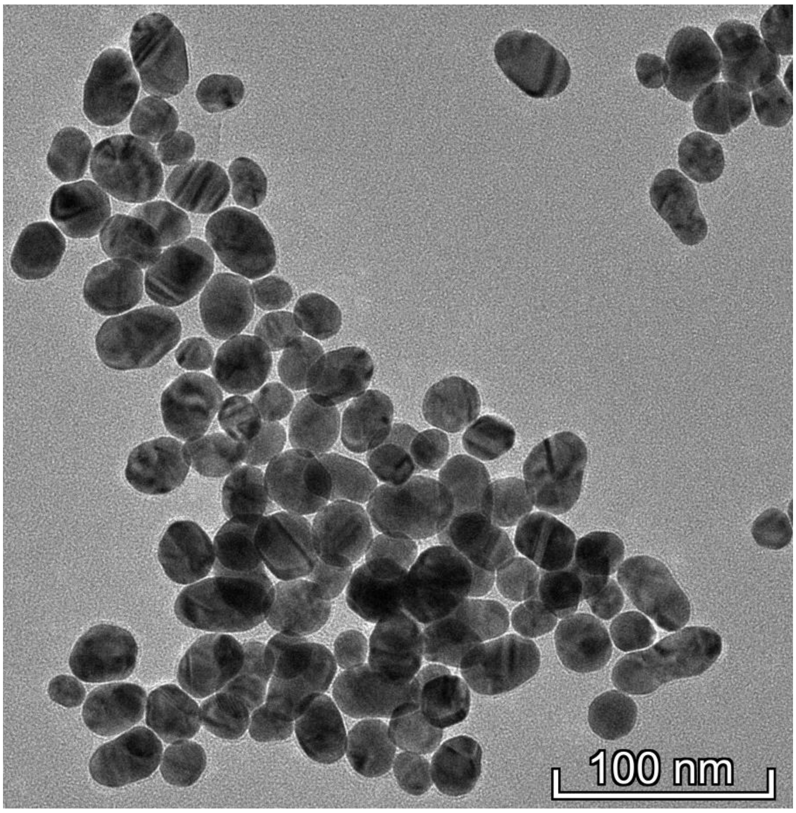

Figure S6. TEM image of the synthesized AuNPs.

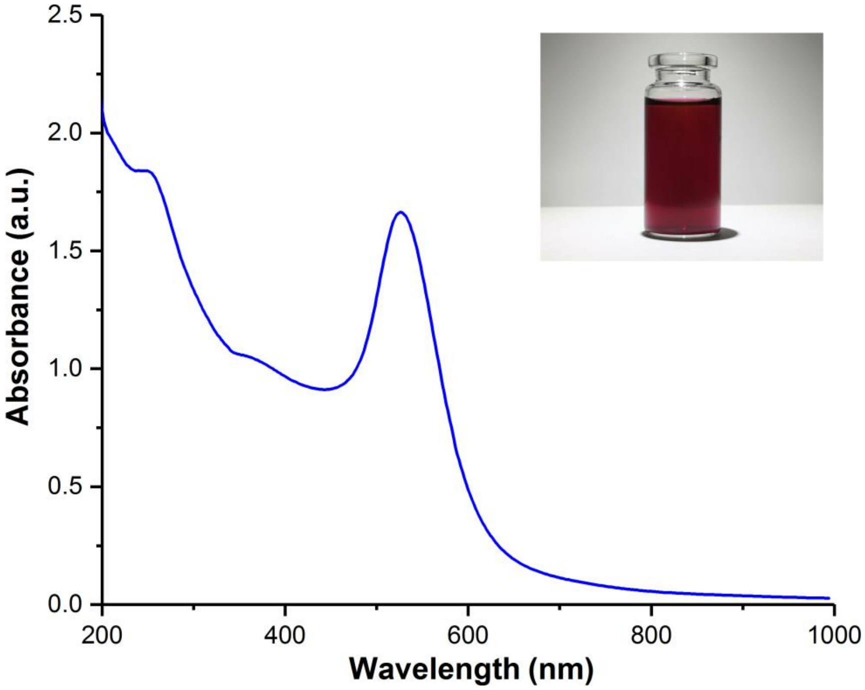

Figure S7. UV-Vis spectrum of the synthesized AuNPs.

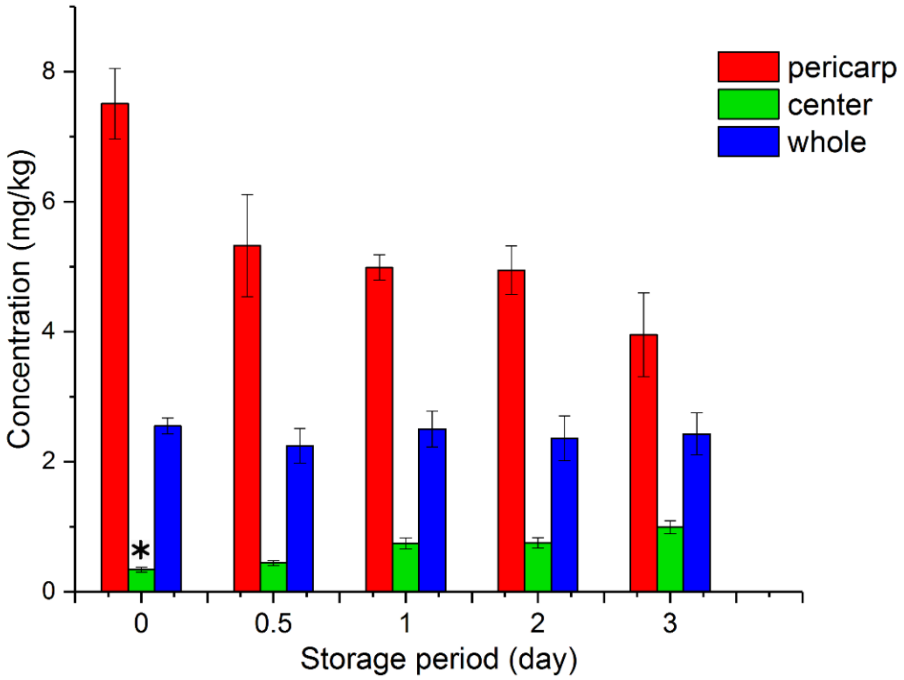

Figure S8. HPLC results of chlorantraniliprole concentration in the pericarp, flesh, and entirety of apples at different times after the application of chlorantraniliprole. The footnote (*) represents the chlorantraniliprole concentration in the center region at day 0 was magnified by 10 times.

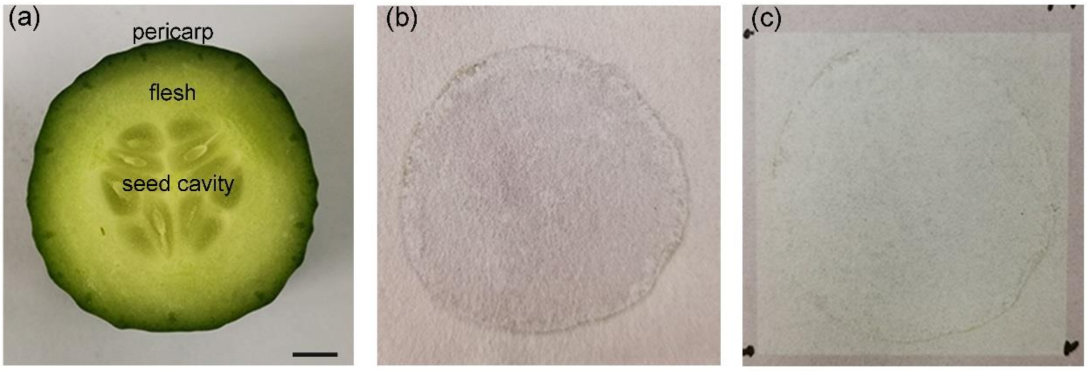

Figure S9. Optical images of (**a**) a cucumber slice sprayed with pesticide, and a AuNP-immersed filter paper imprint of a cucumber slice (**b**) before and (**c**) after laser irradiance. Black dots were used to mark the location of the imaging area. Scale bar, 5 mm.

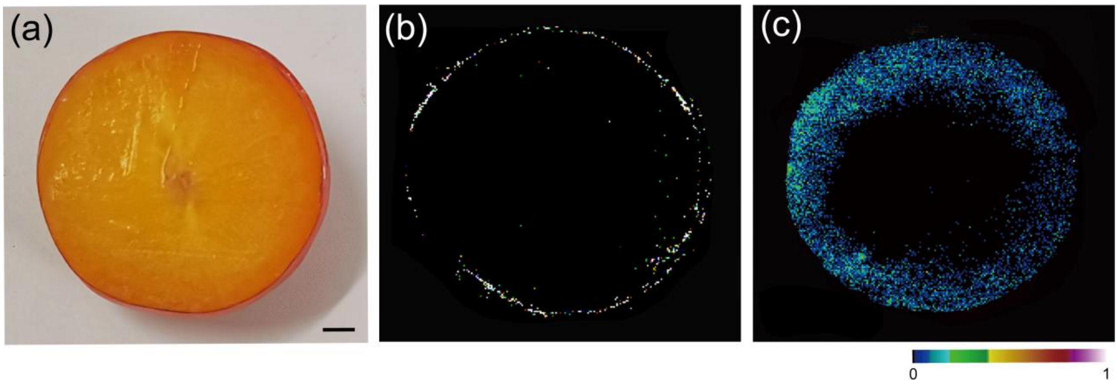

Figure S10. (**a**) Optical and corresponding MS images of a pulp (**b**) 1 day and (**c**) 2 days after azoxystrobin spraying. Scale bar, 4 mm.

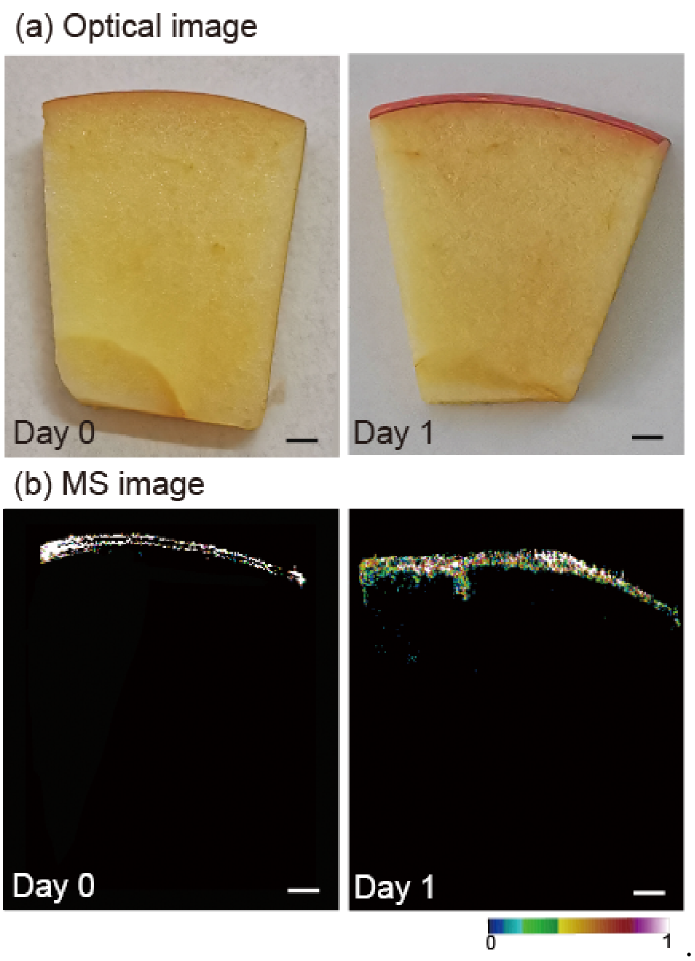

Figure S11. (**a**) Optical and (**b**) MS images of an apple slice 0 days and 1 day after azoxystrobin spraying. Scale bar, 3 mm.

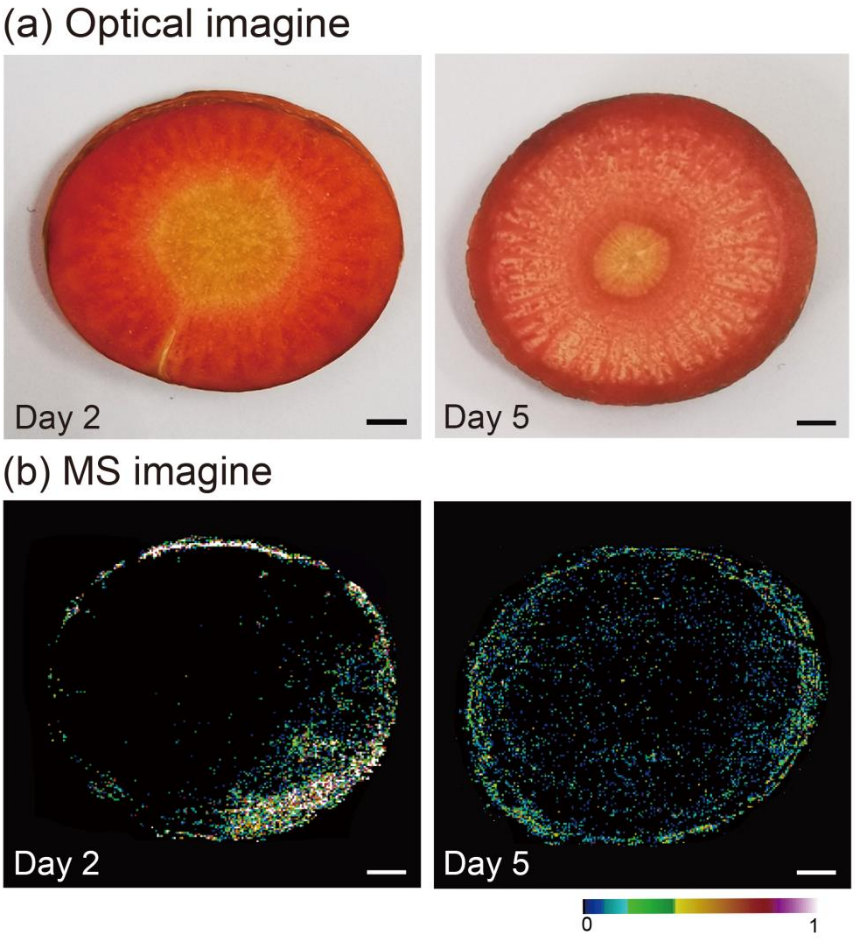

Figure S12. (**a**) Optical and (**b**) MS images of a carrot slice 2 days and 5 day after chlorantraniliprole spraying. Scale bar, 2 mm.
